# Complement C5 inhibition in generalized myasthenia gravis is associated with improved survival and increased cardiovascular risk

**DOI:** 10.3389/fimmu.2026.1812643

**Published:** 2026-05-14

**Authors:** Carl Vahldieck, Benedikt Fels

**Affiliations:** 1 Department of Anesthesiology and Intensive Care Medicine, University Medical Centre Schleswig-Holstein, Luebeck, Germany; 2 Institute of Physiology, University of Luebeck, Luebeck, Germany; 3 DZHK (German Research Centre for Cardiovascular Research), Partner Site North, Luebeck, Germany

**Keywords:** cardiovascular outcomes, complement C5 inhibition, eculizumab, generalized myasthenia gravis, ravulizumab, TriNetX

## Abstract

**Background:**

Complement C5 inhibitors are effective disease-modifying therapies for acetylcholine receptor antibody-positive generalized myasthenia gravis (MG), but cardiovascular safety has not been evaluated as a dedicated outcome domain. With increasing and prolonged use, systematic assessment of cardiovascular and thromboembolic risk - including potential differences between individual C5 inhibitors - is needed.

**Methods:**

We performed a retrospective cohort study using the TriNetX federated electronic health record network, including adults with generalized MG. Propensity score-matched cohorts compared patients treated with C5 inhibitors with untreated controls (N = 1,094 vs. 1,094), and ravulizumab with eculizumab (N = 330 vs. 330). Outcomes included major adverse cardiovascular events (MACE), thrombotic disorders, acute kidney injury (AKI), arrhythmias, and all-cause mortality over 365 days. Associations were evaluated using risk ratios (RR) and Cox proportional hazards models.

**Results:**

After matching, C5 inhibition was associated with increased risk of AKI (6.1% vs 3.2%, p<0.01; RR 1.70) and thrombotic disorders (5.1% vs 2.6%, p=0.002; RR 1.74) compared to no C5-inhibition treatment. All-cause mortality was significantly lower among C5-treated patients after one year (RR 0.54; p=0.045). MACE occurred more frequently with C5 inhibition (8.3% vs 5.5%; p=0.009), with a trend toward higher hazard over time. In agent-specific analyses (ravulizumab vs. eculizumab), ravulizumab was associated with a significantly lower hazard of MACE compared with eculizumab (6.6% vs 11.1%, p=0.041; HR 0.58, while hazards for mortality, thrombotic events, and other cardiovascular outcomes were similar.

**Conclusions:**

C5 inhibition in generalized MG is associated with improved survival but increased cardiovascular and thromboembolic risk. Although limited by its retrospective design, this analysis highlights a clinically relevant safety signal and suggests heterogeneity within the C5 inhibitor class. As use of complement inhibition expands, careful longitudinal cardiovascular monitoring will become increasingly important for clinicians managing patients with MG.

## 1 Introduction

Generalized myasthenia gravis (MG) is a chronic autoimmune neuromuscular disorder characterized by antibody-mediated impairment of neuromuscular transmission, resulting in fluctuating skeletal muscle weakness and fatigability (1). In most patients, pathogenic autoantibodies against the acetylcholine-receptor activate the terminal complement cascade, leading to structural damage of the postsynaptic membrane and sustained disease activity (2, 3). These pathophysiological insights have established inhibition of complement component 5 (C5) as a targeted and disease-modifying therapeutic strategy in acetylcholine receptor (AChR) antibody-positive MG.

However, MG is increasingly recognized as a systemic autoimmune disease characterized by chronic immune activation and systemic inflammatory signalling. Elevated circulating inflammatory cytokines and immune activation markers have been described in patients with MG and are associated with disease activity and immune dysregulation. In autoimmune diseases in general, chronic systemic inflammation has been linked to increased cardiovascular and thrombotic risk, likely mediated through endothelial dysfunction, prothrombotic pathways, and accelerated atherosclerosis. This systemic inflammatory context may therefore be relevant when evaluating cardiovascular outcomes in patients with MG receiving long-term immunomodulatory therapies (4–8).

Eculizumab and the long-acting C5 inhibitor ravulizumab have demonstrated substantial efficacy in patients with refractory generalized MG and are approved for clinical use in this population (9–11). Randomized clinical trials and extension studies, including REGAIN and CHAMPION MG, have shown durable improvements in disease severity and quality of life, with overall favourable safety profiles in trial-selected cohorts (9, 12). Recently, long-term results from the CHAMPION MG open-label extension -the largest available safety dataset for ravulizumab in MG - confirmed sustained efficacy and general tolerability over extended follow-up (12).

In parallel, real-world studies have increasingly informed routine clinical practice. Observational cohorts and registry-based analyses have reported consistent clinical effectiveness of eculizumab and favourable outcomes following transition to ravulizumab, reflecting real-world treatment patterns and persistence (13–17). Pharmacovigilance-based investigations and comparative real-world studies of targeted biologics have further characterized overall adverse event profiles of C5 inhibitors in MG (18–20). Collectively, these data support the expanding use of complement inhibition as a cornerstone of modern MG therapy.

However, despite this growing body of evidence, cardiovascular safety has not been evaluated as a dedicated outcome domain. Existing clinical trials and real-world studies have primarily focused on neuromuscular efficacy and general tolerability, while cardiovascular and thromboembolic events are typically reported only as aggregated adverse events, without standardized definitions, time-to-event analyses, or appropriate comparator cohorts (13–16, 18, 19). Importantly, no prior study has systematically assessed major adverse cardiovascular events (MACE) or thrombotic outcomes in MG patients receiving C5 inhibitors, nor has any investigation directly compared eculizumab and ravulizumab with respect to cardiovascular risk.

This gap is clinically relevant. In other complement-mediated disorders, including paroxysmal nocturnal haemoglobinuria (PNH) and atypical haemolytic uremic syndrome (aHUS), real-world studies have demonstrated persistent thromboembolic and vascular risks despite effective complement blockade, underscoring the importance of indication-specific cardiovascular safety assessments (21, 22). Whether similar risks exist in MG - a disease without primary endothelial complement injury - remains unknown.

Given the increasing and long-term use of C5 inhibitors in MG, a rigorous evaluation of cardiovascular outcomes is therefore warranted. Utilization of large electronic health record networks such as TriNetX enables assessment of incidence rates of MACE and thromboembolic outcomes in patients treated with C5 inhibitors compared with suitable matched control cohorts. This study therefore aims to evaluate the cardiovascular safety of eculizumab and ravulizumab in patients with MG, focusing on cardiovascular events in treated versus untreated populations, and direct comparisons between the two agents. The findings will address a clinically relevant evidence gap and inform clinicians and regulators on the cardiovascular risk profile of long-term complement inhibition in MG.

## 2 Methods

### 2.1 Data source and study design

We performed a retrospective cohort study using the TriNetX Analytics Network, a federated platform of de-identified electronic health records derived from academic and community healthcare organizations. The network captures longitudinal data on diagnoses, prescriptions, laboratory measurements, procedures, and vital status. Data were available from the earliest records in the network until the end of data availability with a data cut-off in January 2026, and all eligible patients recorded during this period were included in the analysis.

Adults aged 18 years or older with a diagnosis of MG were included. MG was defined by recorded ICD-10 diagnosis code G70.0. To ensure adequate characterization of baseline clinical status, patients were required to have at least six months of observable medical history prior to cohort entry.

Patients were classified into treatment groups based on exposure to complement C5 inhibitors eculizumab or ravulizumab identified via the Healthcare Common Procedure Coding System (HCPCS: J1303/J1300/C9052), RXNORM (591781/2107301) or ICD-10 Procedure Coding System (PCS: XW033C6/XW043C6). For analysis comparing C5 inhibitor-treated patients with untreated controls, individuals receiving either agent were pooled into a single C5 inhibitor cohort. The index date for treated patients was defined as the date of first recorded C5 inhibitor use.

A control cohort was constructed from patients with MG who had no recorded exposure to eculizumab or ravulizumab at any time during follow-up. Control patients were assigned a pseudo-index date selected from MG-related encounters, frequency-matched to the calendar-time distribution of treatment initiation in the C5 inhibitor cohort to minimize temporal bias.

To ensure that cardiovascular outcomes reflected incident rather than pre-existing disease, patients were excluded if they had any of the following diagnoses prior to the index date: Acute myocardial infarction (ICD-10: I21, I22), Ischemic stroke (I63), Intracerebral haemorrhage (I61), Pulmonary embolism (I26), Deep vein thrombosis or other venous thromboses (I82). Patients with missing age or insufficient baseline follow-up were also excluded.

**Figure 1:**
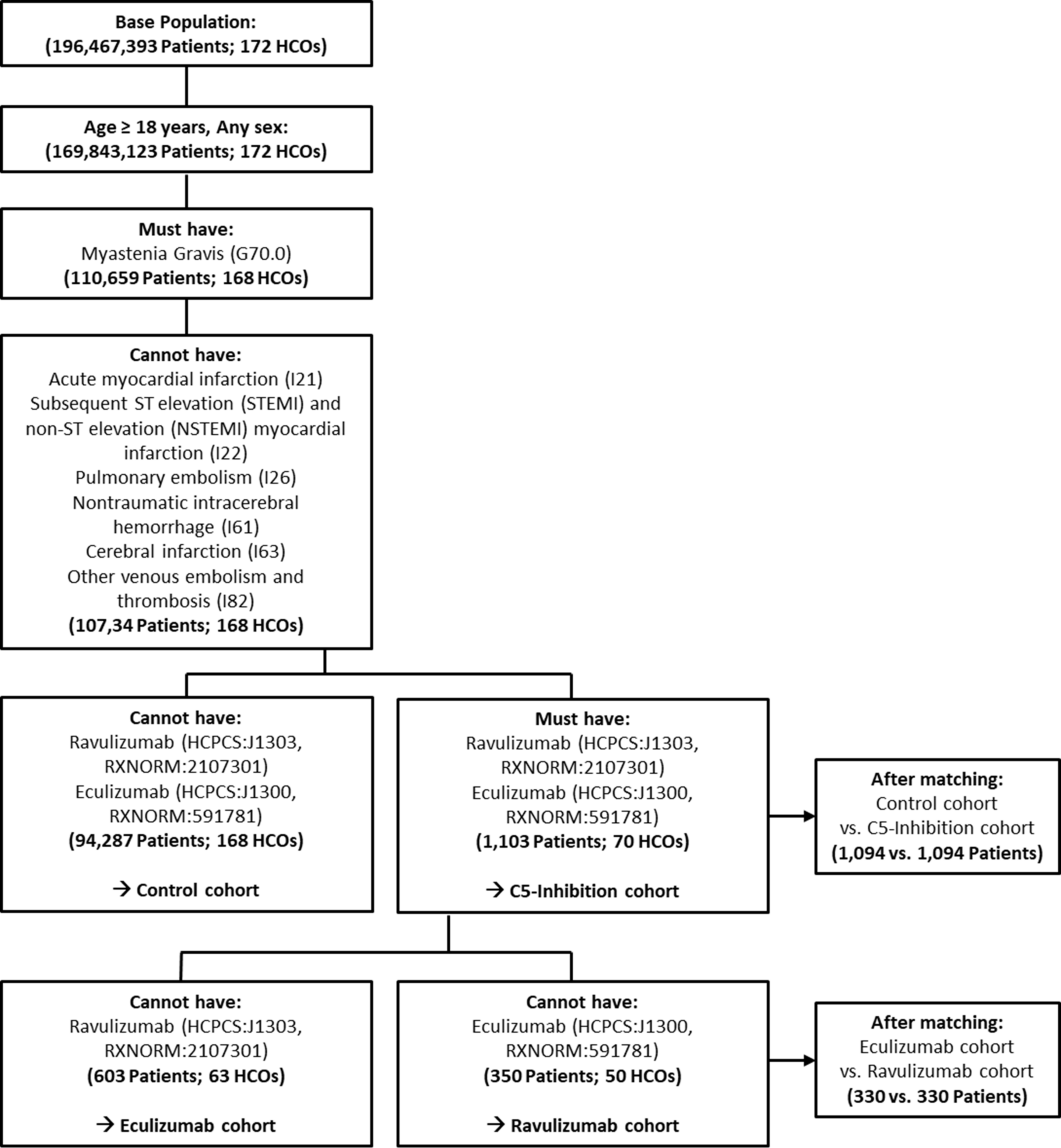
Cohort selection and propensity score-matched study populations. Flow diagram illustrating the identification of patients with generalized myasthenia gravis from the TriNetX network, application of inclusion and exclusion criteria, and construction of analytic cohorts. Patients receiving complement C5 inhibitors and matched control patients without C5 inhibition were identified for class-level comparisons, and separate cohorts of ravulizumab- and eculizumab-treated patients were defined for agent-specific analyses. After exclusion of patients with prior cardiovascular or thromboembolic events and insufficient baseline data, propensity score matching generated balanced propensity score-matched cohorts for each comparison. Final matched sample sizes are shown for all study groups.

Following, we conducted a comprehensive real-world analysis of cardiovascular safety in patients with MG treated with eculizumab or ravulizumab with the dual aim of evaluating both, class-level (MG patients with C5-inhibition vs. without C5-inhibition treatment) as well as agent-specific (MG patients with eculizumab treatment vs. ravulizumab treatment) cardiovascular outcomes (**Figure 1**).

### 2.2 Outcome definitions

Patients were followed from the index date until occurrence of an outcome event, death, last recorded encounter, or end of data availability. Endpoints were defined by clinically relevant combinations of ICD-10 codes (composite endpoints) selected prior to data collection. The ICD-10 codes used are represented in **Table 1**.

**Table 1:** Definitions of clinical endpoints investigated in the study.

Outcomes		ICD-10 codes
Acute Kidney Injury	Acute Kidney Failure	N17
Acute myocardial infarction	Acute myocardial infarction Subsequent ST elevation (STEMI) and non-ST elevation (NSTEMI) myocardial infarction	I21 I22
Cardiac arrhythmias	Paroxysmal tachycardia Atrial fibrillation and flutter Other cardiac arrhythmias	I47 I48 I49
Cardiomyopathy	Ischemic cardiomyopathy Unspecified systolic (congestive) heart failure Unspecified diastolic (congestive) heart failure	I25.5 I50.20 I50.30
Heart failure	Heart failure	I50
Ischemic stroke	Cerebral infarction Sequelae of cerebral infarction	I63 I69.3
Major Adverse Cardiovascular Events (MACE)	Acute myocardial infarction Subsequent ST elevation (STEMI) and non-ST elevation (NSTEMI) myocardial infarction Pulmonary embolism Cardiac arrest Cerebral infarction Sequelae of cerebral infarction Arterial embolism and thrombosis Other venous embolism and thrombosis Cardiogenic shock	I21 I22 I26 I46 I63 I69.3 I74 I82 R57.0
Mortality	Deceased (all-cause death)	R99
Pulmonary embolism	Pulmonary embolism	I26
Thrombotic disorders	Pulmonary embolism Arterial embolism and thrombosis Other venous embolism and thrombosis	I26 I74 I82
Valve disorders	Nonrheumatic aortic valve disorders Nonrheumatic mitral valve disorders Nonrheumatic tricuspid valve disorders Nonrheumatic pulmonary valve disorders	I34 I35 I36 I37
Venous thrombosis	Other venous embolism and thrombosis	I82

### 2.3 Propensity-score matching and statistical analysis

Propensity-score matching (PSM) is a statistical technique that is employed in observational studies to match cohorts in order to equalise the distribution of potential confounding covariates in case and control groups. It is evident that a number of preceding studies have utilised this approach with a view to minimising potential bias. It can thus be concluded that PSM may offer certain advantages in comparison with traditional covariate adjustment (23). Here, PSM was performed for each sub-analysis by establishing a covariate matrix including demographic and clinical data (**Tables 2**, **3**).

**Table 2:** Baseline characteristics and covariate balance before and after propensity score matching in the c5-inhibition and control cohorts.

	Before matching				After matching			
	C5-inhibition (N = 1,103)	Control (N = 94,287)	p-value	Standardized difference	C5-inhibition (N = 1,094)	w/o C5-inhibition (N = 1,094)	p-value	Standardized difference
Age at Index (years)	57.3 +/- 18.1	60.0 +/- 18.0	**<0.001**	0.145	57.3 +/- 18.1	57.8 +/- 18.1	0.586	0.023
Sex (Female)	50.8%	53.4%	0.090	0.052	50.8%	50.9%	0.923	0.004
Ethnicity	73.4%	60.4%	**<0.001**	0.280	73.4%	73.6%	0.966	0.002
Essential (primary) hypertension	23.4%	22.7%	0.571	0.017	23.4%	23.9%	0.801	0.011
Diabetes mellitus	12.2%	10.3%	**0.033**	0.062	12.2%	12.3%	0.948	0.003
Chronic kidney disease	4.3%	4.3%	0.952	0.002	4.3%	3.7%	0.514	0.028
Disorders of lipoprotein metabolism and other lipidaemia’s	48.5%	51.1%	0.5	0.05	49.1%	49.6%	0.91	0.010
Personal history of nicotine dependence	28.3%	18.2%	**<0.001**	0.24	29.0%	29.6%	0.92	0.010
Nicotine dependence	21.0%	10.2%	**<0.001**	0.29	20.4%	19.3%	0.8	0.030
Atrial fibrillation and flutter	2.6%	4.5%	**0.002**	0.107	2.6%	2.1%	0.479	0.030
Chronic ischemic heart disease	6.8%	7.3%	0.485	0.022	6.8%	5.9%	0.379	0.038
Transfusion	0.9%	0.7%	0.320	0.028	0.9%	0.9%	1	<0.001
Warfarin	2.2%	2.1%	0.826	0.007	2.2%	2.1%	0.883	0.006
Rivaroxaban	1.6%	1.3%	0.459	0.022	1.6%	1.3%	0.587	0.023
Apixaban	2.7%	2.2%	0.253	0.033	2.7%	3.0%	0.701	0.016
Dabigatran	0%	<0.1%	0.622	0.021	0%	0%	--	--
Edoxaban	0%	<0.1%	0.486	0.030	0%	0.9%	0.002	0.136

The bold values indicate statistically significant results.

**Table 3:** Baseline characteristics and covariate balance before and after propensity score matching in the ravulizumab and eculizumab cohorts.

	Before matching				After matching			
	Ravulizumab (N = 350)	Eculizumab (N = 603)	p-value	Standardized difference	Ravulizumab (N = 330)	Eculizumab (N = 330)	p-value	Standardized difference
Age at Index (years)	60.5 ± 17	56.7 ± 18	**0.0014**	0.2184	59.8 ± 17	59.2 ± 17.1	0.6500	0.035
Sex (Female)	45.8	54.5	**0.0099**	0.1744	47.6	47.6	1.0000	<0.001
Ethnicity	75.9	72.1	0.2032	0.0864	74.8	47.6	0.6504	0.035
Essential (primary) hypertension	28.6	21.6	**0.0152**	0.1621	26.1	23.3	0.4166	0.063
Diabetes mellitus	12.6	13.1	0.8319	0.0143	12.4	10.9	0.5443	0.047
Chronic kidney disease	4.9	4.0	0.5387	0.0410	4.2	3.0	0.4055	0.065
Disorders of lipoprotein metabolism and other lipidaemia’s	47.2%	49.2%	0.5	0.05	47.1%	47.6%	0.91	0.010
Personal history of nicotine dependence	28.3%	27.2%	0.6	0.04	27.0%	26.6%	0.81	0.02
Nicotine dependence	25.0%	20.2%	**0.041**	0.29	20.4%	19.3%	0.8	0.030
Atrial fibrillation and flutter	3.4	2.5	0.4118	0.0542	3.0	3.0	1.0000	<0.001
Chronic ischemic heart disease	10.0	6.0	**0.0248**	0.1471	8.2	7.2	0.6619	0.034
Transfusion	2.9	1.7	0.2209	0.0798	3.0	3.0	1.0000	<0.001
Warfarin	3.4	1.6	0.0832	0.1117	3.0	3.0	1.0000	<0.001
Rivaroxaban	2.8	1.7	0.2209	0.0798	3.0	3.0	1.0000	<0.001
Apixaban	3.7	2.2	0.1615	0.0913	3.0	3.0	1.0000	<0.001
Dabigatran	0	0	–	–	0	0	–	–
Edoxaban	0	0	–	–	0	0	–	–

The bold values indicate statistically significant results.

Baseline characteristics included: age at index, sex, ethnicity, diabetes mellitus (E08-E13), overweight and obesity (E66), disorders of lipoprotein metabolism and other lipidaemia’s (E78), essential (primary) hypertension (I10), chronic ischemic heart disease (I25), atrial fibrillation and flutter (I48), nicotine dependence (F17) or personal history of nicotine dependence (Z87.891), chronic kidney disease (N18), history of transfusion of blood or blood components (36430), therapy with warfarin (11289), dabigatran (1546356), rivaroxaban (1114195), apixaban (1364430) or edoxaban (1599538). These variables were selected *a priori* based on their known associations with cardiovascular risk (**Tables 2**, **3**).

Patient order was randomized before PSM to prevent order-dependent matching bias. A propensity-score for each patient was generated by logistic regression and matching was performed 1:1 using the greedy nearest neighbour approach with a calliper distance of 0.1 using the TriNetX analysis software. Baseline characteristics were re-evaluated and reported after matching, differences were compared by t-test. Continuous variables were assumed to be approximately normally distributed. Given the large sample size and the robustness of t-tests to moderate deviations from normality, parametric tests were used for group comparisons, and non-parametric tests were not required.

Following PSM cardiac and vascular endpoints and composite endpoints (**Table 1**) as well as all-cause death for class-level and agent-specific analyses were evaluated. Odds Ratio (OR), Risk Ratio (RR) and Risk Difference (RD) were calculated. Time-to-event outcomes were analysed using Kaplan-Meier survival curves and Cox proportional hazards regression models to estimate Hazard Ratio (HR). The proportional hazard assumption was tested using the generalized Schoenfeld approach built in the TriNetX platform. In all the analyses, a 95% confidence interval (95% CI) was considered evidence of statistical significance.

To illustrate the magnitude of group differences, results were converted into Cohen’s D effect sizes (effect size of d ≥ 0.20 to d < 0.50 was considered a small effect, d ≥ 0.50 to d < 0.80 was considered a medium effect, and d ≥ 0.80 was considered a large effect). Statistical significance was defined as p-value <0.05. Graphs were created using GraphPad Prism (GraphPad Software Inc., Boston, MA).

### 2.4 Ethical statement

This study was performed using data obtained from the TriNetX federated research network, which exclusively provides aggregate-level, de-identified patient information in compliance with the de-identification requirements specified in §164.514(a) of the Health Insurance Portability and Accountability Act (HIPAA) Privacy Rule (24). The analysis represents a secondary use of previously collected data and does not involve any form of clinical intervention or direct contact with patients. Data anonymization within TriNetX is ensured through a formal expert determination process, as outlined in §164.514(b) of the HIPAA Privacy Rule (24). The Ethics Committee of the University of Lübeck confirmed that investigations based solely on TriNetX data do not require additional ethical review or approval. As the dataset contains no individual-level identifiers and excludes any interaction with human participants, the analyses qualify as research using de-identified secondary data and are therefore exempt from informed consent requirements. This determination is consistent with prior publications from the University of Lübeck and the University Hospital Schleswig-Holstein (25), which have similarly concluded that studies relying on de-identified secondary datasets do not constitute human subjects research and thus fall outside the scope of ethics committee oversight.

The University Hospital Schleswig-Holstein (Luebeck, Germany) is an active healthcare organization within the TriNetX network, ensuring that all institutional, regulatory, and legal standards governing the use of de-identified patient data are fulfilled. Reporting of this study followed the recommendations of the STROBE-statement (STrengthening the Reporting of OBservational studies in Epidemiology) for observational research.

## 3 Results

### 3.1 Cohort description and baseline characteristics: C5-inhibition cohort vs control cohort

Prior to PSM, the analytic cohorts used for outcome analyses included 1,103 C5-treated patients (C5-inhibition cohort) and 94,287 untreated controls (control cohort) (**Table 2**). Several baseline characteristics differed significantly between groups. Patients receiving C5 inhibition were younger (57.3 vs 60.0 years, p<0.001), more frequently of the predominant ethnicity (73.4% vs 60.4%, p<0.001), had a higher prevalence of type 2 diabetes (12.2% vs 10.3%, p=0.033), more often a personal history of nicotine dependence or ongoing nicotine dependence (both p<0.001) and a lower prevalence of atrial fibrillation (2.6% vs 4.5%, p=0.002). After PSM, 1,094 patients remained in each cohort, and all previously significant imbalances were eliminated, indicating excellent covariate balance (**Table 2**).

### 3.2 Cardiovascular and thromboembolic outcomes: C5-inhibition cohort vs control cohort

**Figure 2:**
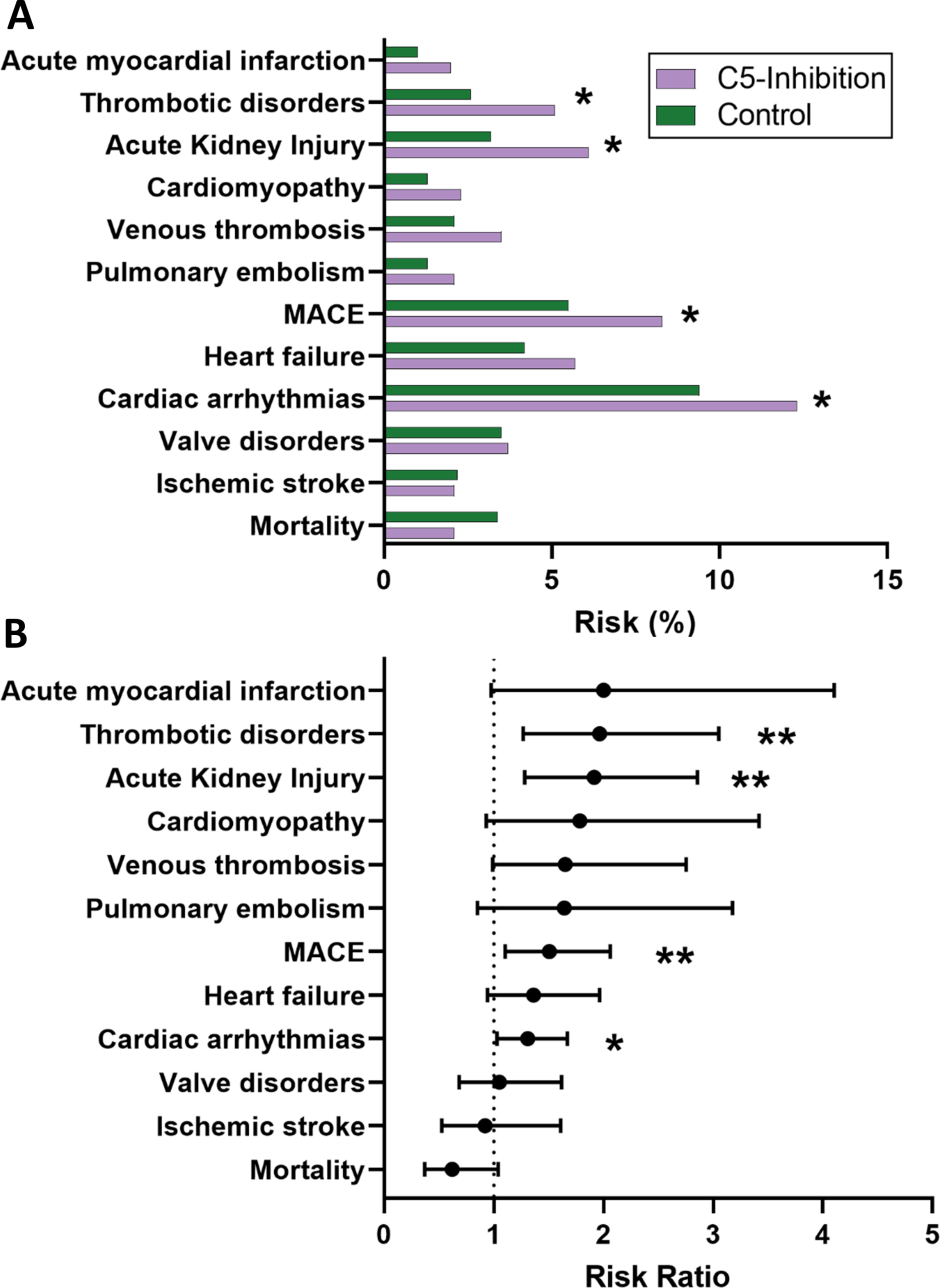
Cardiovascular and thromboembolic outcomes in propensity score-matched myasthenia gravis cohorts with and without C5 inhibition. **(A)** Absolute 1-year event rates for cardiovascular and thromboembolic outcomes in propensity score-matched patients with myasthenia gravis treated with C5 inhibitors compared with matched control patients without C5 inhibition. **(B)** Corresponding risk ratios with 95% confidence intervals for each outcome in the propensity score-matched cohorts. C5 inhibitor treatment was associated with higher risks of thrombotic disorders, acute kidney injury, cardiomyopathy, pulmonary embolism, and major adverse cardiovascular events, while all-cause mortality did not differ significantly between groups. The vertical line indicates no difference to between treatments (risk ratio = 1). Asterisks indicate statistically significant differences between cohorts (p<0.05).

During the 365-day follow-up, patients treated with C5 inhibitors exhibited significantly higher risks for several cardiovascular and thromboembolic outcomes (**Table 4**). The risk of AKI was significantly higher in the C5-inhibition cohort (6.1% vs 3.2%; OR 1.974, p<0.001). Cardiac arrhythmias were more frequent (12.3% vs 9.4%; OR 1.354, p=0.028). Thrombotic disorders occurred in 5.1% of C5-treated patients compared with 2.6% of controls (OR 2.018, p=0.002) (**Figure 2A**).

**Table 4:** Cardiovascular outcomes of Myastenia Gravis patients with vs. without C5-Inhibition therapy.

Outcome	C5-inhibition (N = 1094)		w/o C5-inhibition (N = 1094)										
	N of outcomes	Risk (%)	N of outcomes	Risk (%)	Risk ratio	RR 95% CI	Odds ratio	OR 95% CI	Risk difference	RD 95% CI	z	p-value	Cohen’s D
Acute Kidney Injury	67	6.1	35	3.2	1.914	(1.283, 2.856)	1.974	(1.300, 2.997)	0.029	(0.012, 0.047)	3.245	**<0.001**	0.36
Acute myocardial infarction	22	2.0	11	1.0	2	(0.975, 4.104)	2.021	(0.975, 4.187)	0.010	(-0.000, 0.020)	1.929	0.054	0.39
Cardiac arrhythmias	135	12.3	103	9.4	1.311	(1.029, 1.670)	1.354	(1.033, 1.777)	0.029	(0.003, 0.055)	2.197	**0.028**	0.15
Cardiomyopathy	25	2.3	14	1.3	1.786	(0.933, 3.417)	1.804	(0.933, 3.490)	0.010	(-0.001, 0.021)	1.777	0.076	0.32
Heart failure	64	5.7	47	4.2	1.362	(0.943, 1.966)	1.384	(0.941, 2.036)	0.015	(-0.003, 0.033)	1.655	0.098	0.17
Ischemic stroke	23	2.1	25	2.2	0.920	(0.525, 1.611)	0.918	(0.518, 1.628)	-0.002	(-0.014, 0.010)	-0.292	0.770	-0.05
MACE	92	8.3	61	5.5	1.508	(1.103, 2.062)	1.554	(1.112, 2.172)	0.028	(0.007, 0.049)	2.597	**0.009**	0.23
Mortality	23	2.1	37	3.4	0.622	(0.372, 1.039)	0.613	(0.362, 1.040)	-0.013	(-0.026, 0.001)	-1.833	0.067	-0.26
Pulmonary embolism	23	2.1	14	1.3	1.643	(0.850, 3.176)	1.657	(0.848, 3.237)	0.008	(-0.003, 0.019)	1.492	0.136	0.27
Thrombotic disorders	57	5.1	29	2.6	1.966	(1.267, 3.050)	2.018	(1.280, 3.180)	0.025	(0.009, 0.041)	3.079	**0.002**	0.37
Valve disorders	41	3.7	39	3.5	1.051	(0.684, 1.617)	1.053	(0.674, 1.646)	0.002	(-0.014, 0.017)	0.228	0.820	0.03
Venous thrombosis	38	3.5	23	2.1	1.652	(0.991, 2.754)	1.676	(0.991, 2.832)	0.014	(-0.000, 0.027)	1.948	0.051	0.28

MACE: Major Adverse Cardiovascular Events; CI, confidence interval; RR, risk Ratio; RD, Risk Difference; OR, Odds Ratio; w/o, without.Data showing Risk Ratio, Odds Ratio and Risk Difference with 95%-Confidence Intervals and effect size (Cohen’s D).The bold values indicate statistically significant results.

The composite endpoint MACE occurred in 8.3% of C5-treated patients compared with 5.5% of controls, corresponding to a significantly increased risk (OR 1.554, p=0.009). The difference in the composite MACE endpoint appeared to be driven primarily by arterial cardiovascular and thrombotic events, including myocardial infarction and arterial thrombosis, whereas venous thromboembolic events contributed less to the composite endpoint. The absolute risk-increase for MACE associated with C5 inhibition compared with matched controls was approximately 2.8% at one year.

Rates of acute myocardial infarction were numerically higher (2.0% vs 1.0%; OR 2.021, p=0.054) but did not reach statistical significance. Venous thrombosis also showed a borderline increase (3.5% vs 2.1%; OR 1.676, p=0.051). There were no statistically significant differences in ischemic stroke or valve disorders. Mortality tended to be lower in the C5-treated group (2.1% vs 3.4%; OR 0.613, p = 0.067) but did not reach statistical significance (**Figure 2B**).

### 3.3 Time-to-event analyses for cardiovascular and thromboembolic outcomes: C5-inhibition cohort vs control cohort

Kaplan-Meier survival analyses were performed to compare the cumulative incidence of cardiovascular and thromboembolic outcomes between patients with MG treated with C5 inhibitors and matched controls without C5 inhibition (**Table 5**).

**Table 5:** Kaplan Meier-Results of Myastenia Gravis patients with vs. without C5-Inhibition therapy or C5-Inhibition vs Control cohort.

Outcome	C5-Inhibition (N = 1094)		Control (N = 1094)							
	N of outcomes	Survival (%)	N of outcomes	Survival (%)	χ² (log-rank)	p-value	Hazard ratio	HR 95% CI	HR χ²	p-value
Mortality	23	97.8	37	96.1	5.506	**0.019**	0.541	0.322-0.911	4.031	**0.045**
Cardiac arrhythmia	135	87.2	103	89.1	1.371	0.242	1.165	0.902-1.506	0.956	0.328
Acute Kidney Injury	67	93.6	35	96.3	6.658	**0.010**	1.702	1.131-2.562	1.025	0.311
Cardiomyopathy	25	97.6	14	98.5	1.112	0.179	1.432	0.811-3.002	1.079	0.299
Venous thrombosis	38	96.4	23	97.6	1.888	0.169	1.435	0.855-2.409	4.208	**0.040**
Pulmonary embolism	23	97.8	14	98.5	1.113	0.291	1.427	0.734-2.774	2.968	0.085
AMI	22	97.9	11	98.9	2.347	0.126	1.748	0.848-3.606	2.675	0.102
MACE	92	91.4	61	93.7	3.417	**0.045**	1.355	0.981-1.874	1.786	0.181
Ischemic stroke	23	97.9	25	97.4	0.363	0.547	0.840	0.477-1.481	2.467	0.116
Heart failure	64	94.0	47	95.1	0.998	0.318	1.211	0.831-1.765	0.544	0.461
Thrombotic disorders	57	94.7	29	96.9	6.043	**0.014**	1.740	1.112-2.720	1.385	0.239
Valve disorders	41	96.2	39	95.9	0.101	0.750	0.931	0.601-1.444	0.018	0.893

AMI, Acute myocardial infarction; MACE, Major adverse cardiovascular events; HR, Hazard Ratio.The bold values indicate statistically significant results.

**Figure 3:**
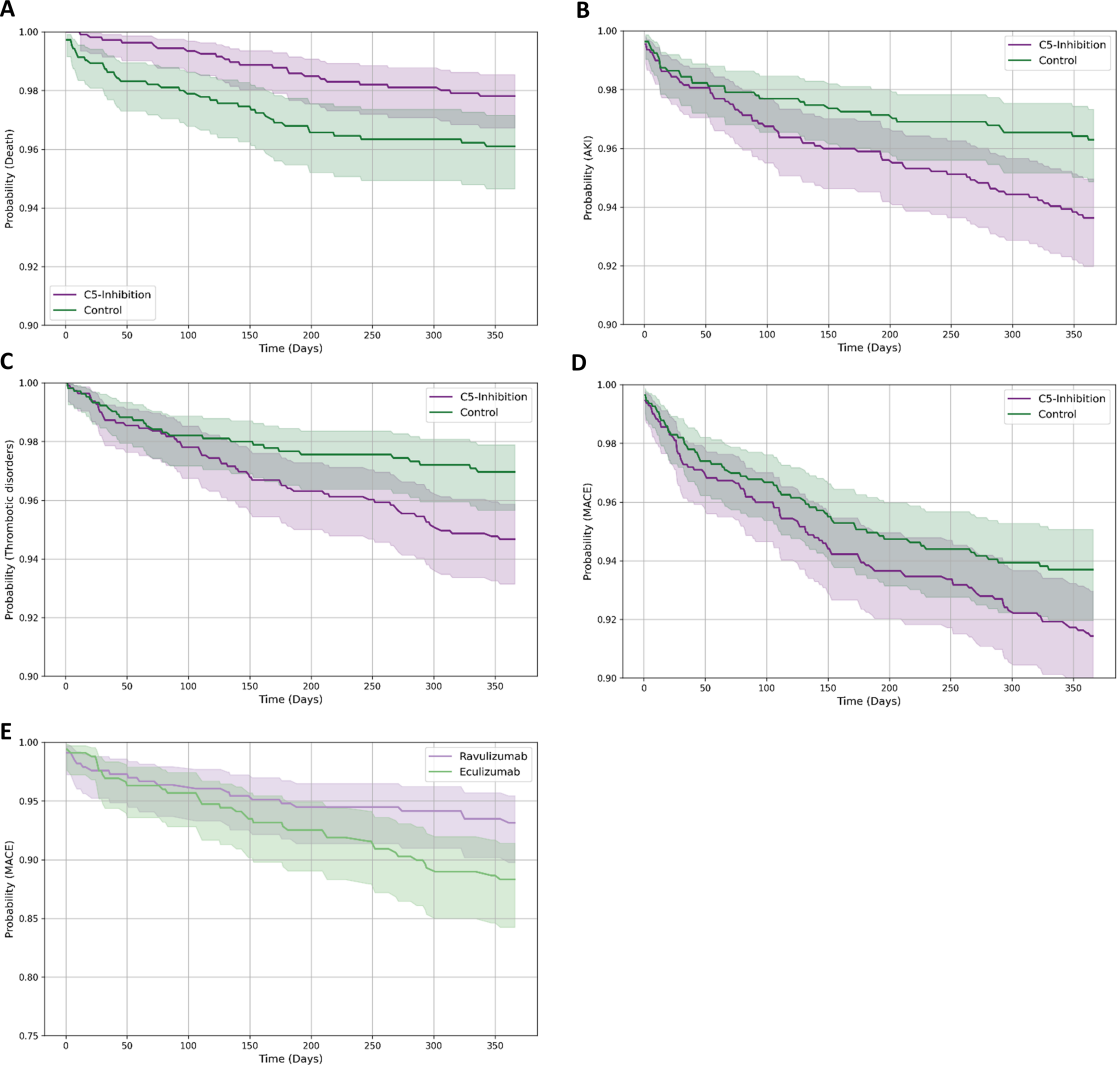
Kaplan-Meier analysis of major adverse cardiovascular events with ravulizumab versus eculizumab in propensity-score-matched myasthenia gravis cohorts. Kaplan-Meier curves showing event-free survival over 365 days in propensity score-matched patients with myasthenia gravis. Panels **(A-D)** compare patients treated with C5 inhibitors with matched control patients without C5 inhibition: **(A)** all-cause mortality, **(B)** acute kidney injury, **(C)** thrombotic disorders, and **(D)** major adverse cardiovascular events. Panel **(E)** shows event-free survival for major adverse cardiovascular events over 365 days in propensity score-matched patients treated with ravulizumab versus eculizumab. Ravulizumab was associated with higher event-free survival and a significantly lower hazard of major adverse cardiovascular events compared with eculizumab.

All-cause mortality (death) was significantly lower in the C5-inhibition cohort compared with controls (**Figure 3A**). The difference was statistically significant by log-rank testing (p=0.019), and Cox regression demonstrated a significantly reduced hazard of death associated with C5 inhibition (HR 0.541, p=0.045).

For AKI (**Figure 3B**), event-free survival was significantly lower in the C5-inhibition cohort (93.6% vs 96.3%). The log-rank test confirmed a significant difference (p=0.010), with a higher hazard of AKI in C5-treated patients (HR 1.702).

Similarly, thrombotic disorders (**Figure 3C**) occurred more frequently in patients receiving C5 inhibitors, with 365-day event-free survival of 94.7% compared with 96.9% in controls. This difference was significant (p=0.014), corresponding to a 74% higher hazard of thrombotic events (HR 1.740).

In contrast, arrhythmias did not differ significantly between groups (87.2% vs 89.1% event-free survival). Likewise, acute myocardial infarction, ischemic stroke, heart failure, valve disorders, and cardiomyopathy showed no statistically significant differences between the cohorts (all log-rank p > 0.10).

For the composite endpoint of MACE (**Figure 3D**), C5-treated patients exhibited lower event-free survival (91.4% vs 93.7%), with a trend toward significance (p=0.065), although the HR did not reach statistical significance (HR 1.355; p=0.181).

Collectively, these time-to-event analyses demonstrate that while C5 inhibition in MG is associated with improved survival, it is also accompanied by a significantly increased risk of AKI and thrombotic complications, with more modest and non-significant trends for several other cardiovascular outcomes.

### 3.4 Cohort description and baseline characteristics: ravulizumab-treated cohort vs eculizumab-treated cohort

Among C5-treated patients, 350 received ravulizumab without prior eculizumab and 603 received eculizumab without ravulizumab (**Table 3**).

Before PSM of the analytic cohorts ravulizumab-treated patients were older (60.5 vs 56.7 years, p=0.0014), more often male (female 45.8% vs 54.5%, p<0.01), had higher rates of hypertension (28.6% vs 21.6%, p=0.015), Nicotine dependence (25.0% vs 20.2%, p=0.041) and chronic ischemic heart disease (10.0% vs 6.0%, p=0.025).

After matching, 330 patients remained in each group, and all baseline imbalances were resolved, including age, sex, hypertension, nicotine dependence and ischemic heart disease.

### 3.5 Cardiovascular and thromboembolic outcomes: ravulizumab-treated vs eculizumab-treated cohorts

In the matched cohorts, ravulizumab was associated with a significantly lower risk of MACE compared with eculizumab (6.6% vs 11.1%; OR 0.566, p = 0.041, Cohen’s d = −0.29) (**Table 6**).

**Table 6:** Cardiovascular outcomes of Myastenia Gravis patients with Ravulizumab vs. Eculizumab C5-Inhibition therapy.

Outcome	Ravulizumab (N = 330)		Eculizumab (N = 330)										
	N of outcomes	Risk (%)	N of outcomes	Risk (%)	Risk ratio	RR 95% CI	Odds ratio	OR 95% CI	Risk difference	RD 95% CI	z	p-value	Cohen’s D
Acute Kidney Injury	25	7.6	28	8.5	0.893	(0.532, 1.498)	0.884	(0.504, 1.551)	-0.909	(-5.055, 3.237)	-0.43	0.667	-0.06
Acute myocardial infarction	11	3.3	13	3.9	0.846	(0.385, 1.862)	0.841	(0.371, 1.905)	-0.601%	(-3.431, 2.23)	-0.416	0.677	-0.09
Cardiac arrhythmias	38	11.5	51	15.4	0.745	(0.504, 1.102)	0.712	(0.454, 1.118)	-3.939	(9.142, 1.264)	-1.482	0.139	-0.16
Cardiomyopathy	9	3	9	3	1	(0.422, 2.371)	1	(0.411, 2.435)	0	(-2.616, 2.616)	0	1.000	0
Heart failure	18	5.4	25	7.5	0.721	(0.401, 1.294)	0.704	(0.376, 1.316)	-2.102%	(-5.832, 1.627)	-1.104	0.269	-0.18
Ischemic stroke	6	2	9	3	0.948	(0.584, 1.564)	0.934	(0.540, 1.536)	-0.910	(-3.055, 3.237)	-0.43	0.764	-0.03
MACE	22	6.6	37	11.1	0.595	(0.359, 0.986)	0.566	(0.326, 0.982)	-4.505%	(-4.807, -0.202)	-2.046	**0.041**	-0.29
Mortality	6	2	6	2	1	(0.411, 2.281)	1	(0.391, 2.331)	0	(-2.421, 2.418)	0	1.000	0
Pulmonary embolism	4	1.2	5	1.5	1.021	(0.694, 1.627)	1.033	(0.634, 1.631)	0.002	(-0.140, 0.170)	0.228	0.820	0.01
Thrombotic disorders	9	3	9	3	1	(0.432, 2.362)	1	(0.420, 2.414)	0	(-2.605, 2.621)	0	1.000	0
Valve disorders	9	3	18	5.4	0.556	(0.26, 1.186)	0.542	(0.246, 1.192)	-2.402%	(-5.445, 0.644)	-1.545	0.122	-0.32
Venous thrombosis	5	1.5	4	1.2	1.012	(0.704, 1.711)	1.030	(0.654, 1.652)	0.002	(-0.145, 0.182)	0.228	0.830	0.01

MACE, Major Adverse Cardiovascular Events; CI, confidence interval; RR, risk Ratio; RD, Risk Difference; OR, Odds Ratio. Data showing Risk Ratio, Odds Ratio and Risk Difference with 95%-Confidence Intervals and effect size (Cohen’s D).The bold values indicate statistically significant results.

**Figure 4:**
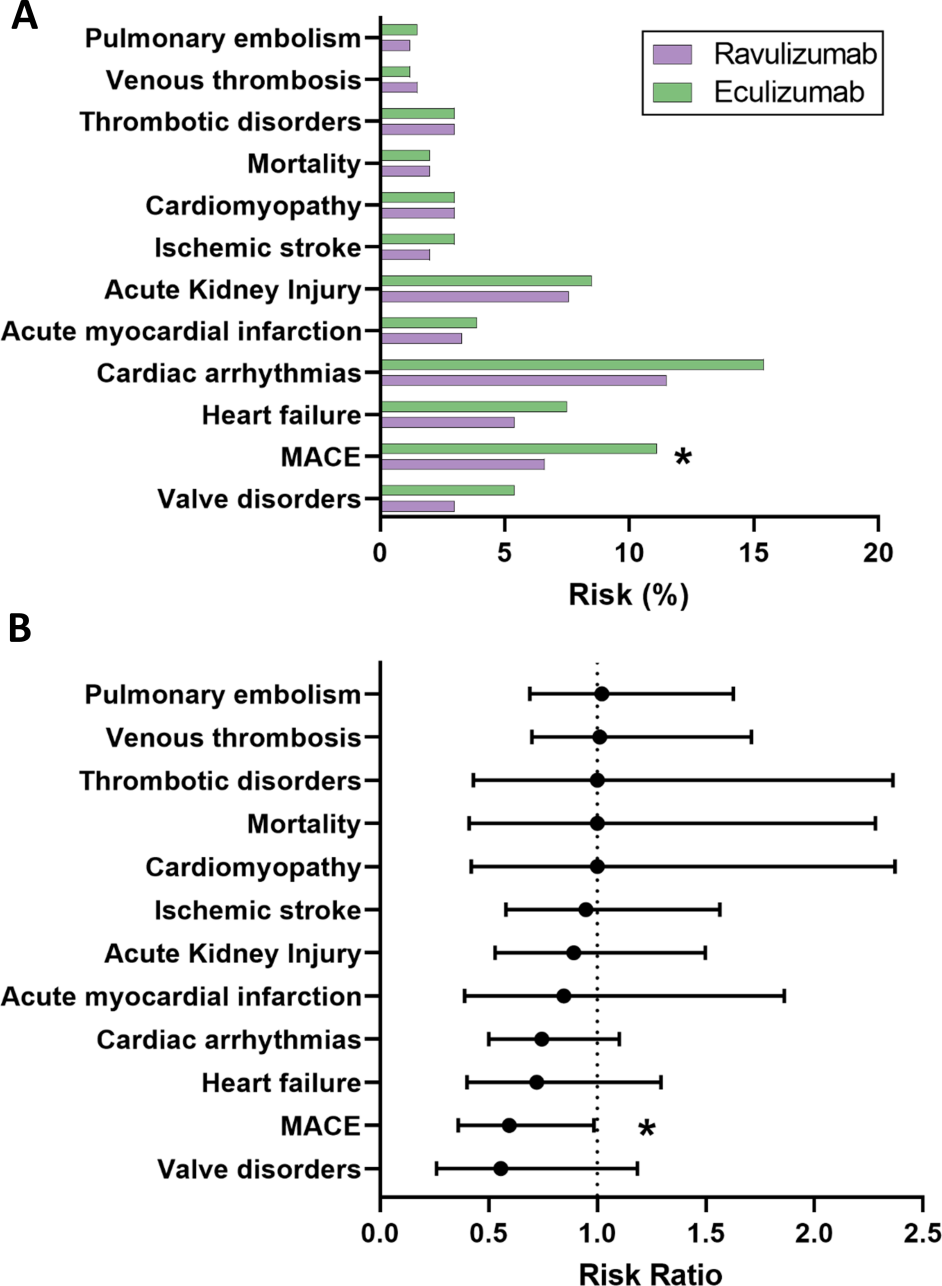
Hazard ratios for cardiovascular and thromboembolic outcomes with ravulizumab versus eculizumab in propensity score-matched myasthenia gravis cohorts. **(A)** Absolute 1-year event rates for cardiovascular and thromboembolic outcomes in propensity score-matched patients with myasthenia gravis treated with ravulizumab compared with matched patients treated with eculizumab. **(B)** Corresponding risk ratios with 95% confidence intervals for each outcome in the propensity score-matched cohorts. Ravulizumab was associated with a significantly lower risk of major adverse cardiovascular events, while risk ratios for other cardiovascular and thromboembolic outcomes did not differ significantly between treatments. The vertical line indicates no difference to eculizumab treatment (risk ratio = 1). Asterisks indicate statistically significant differences between cohorts (p<0.05).

Taken together, these data indicate that while C5 inhibition in MG was associated with increased risks of AKI (6.1%), arrhythmias (12.3%), thrombotic disorders (5.1%), and MACE (8.3%) compared with untreated controls (**Table 4**), ravulizumab demonstrated a significantly more favourable cardiovascular safety profile than eculizumab for the composite MACE endpoint (**Table 6**; **Figures 4A, B**).

No statistically significant differences were observed for AKI, myocardial infarction, arrhythmia, heart failure, or ischemic stroke. Similarly, mortality was identical in both groups. There were no differences in pulmonary embolism, thrombotic disorders, or venous thrombosis.

### 3.6 Kaplan-Meier analysis of cardiovascular and thromboembolic outcomes: ravulizumab-treated cohort vs eculizumab-treated cohort

Time-to-event analyses compared cardiovascular and thromboembolic outcomes between patients with MG treated with ravulizumab and those treated with eculizumab (**Table 7**). Ravulizumab was associated with a significantly lower risk of MACE, with 365-day event-free survival of 93.1% versus 88.3% with eculizumab. Kaplan-Meier curves diverged early and remained separated (log-rank p=0.043), corresponding to a 42% lower hazard (HR 0.584) (**Figure 3E**). No other cardiovascular, renal, or thromboembolic endpoints - including arrhythmia, AKI, heart failure, myocardial infarction, cardiomyopathy, or valve disorders - differed significantly between treatments (all log-rank p>0.10; HR p>0.05). Taken together, these findings indicate a selective reduction in MACE with ravulizumab without differences in other cardiovascular outcomes, consistent with a more favourable cardiovascular risk profile within the C5 inhibitor class in MG.

**Table 7:** Kaplan Meier-Results of Myastenia Gravis patients with Ravulizumab vs. Eculizumab C5-Inhibition therapy.

Outcome	Ravulizumab (N = 330)		Eculizumab (N = 330)							
	N of outcomes	Survival (%)	N of outcomes	Survival (%)	χ² (log-rank)	p-value	Hazard ratio	HR 95% CI	HR χ²	p-value
Arrhythmia	38	88.1	51	83.9	2.059	0.151	0.736	0.484-1.121	1.859	0.173
Acute Kidney Injury	25	92.1	28	91.2	0.146	0.702	0.900	0.525-1.544	0.631	0.427
Cardiomyopathy	25	97.6	14	98.5	1.112	0.179	1.432	0.811-3.002	1.079	0.299
AMI	11	96.5	13	95.9	0.197	0.657	0.834	0.374-1.862	0.146	0.702
MACE	22	93.1	37	88.3	4.093	**0.043**	0.584	0.345-0.990	1.156	0.282
Heart failure	18	94.4	25	92.1	1.201	0.273	0.714	0.390-1.309	3.428	0.064

AMI, Acute myocardial infarction; MACE, Major adverse cardiovascular events; HR, Hazard Ratio.The bold values indicate statistically significant results.

## 4 Discussion

In this large real-world analysis of patients with generalized MG, we provide the first outcomes-focused evaluation of cardiovascular and thromboembolic risk, associated with complement C5 inhibition. Using propensity score-matched cohorts and complementary time-to-event analyses, we demonstrate that treatment with C5 inhibitors is associated with higher rates of AKI, thrombotic disorders, arrhythmias, and MACE compared with untreated MG patients, while being simultaneously associated with lower all-cause mortality over time. Furthermore, direct comparison between the two agents eculizumab and ravulizumab revealed a significantly lower MACE risk with ravulizumab, identifying clinically relevant heterogeneity within the C5 inhibitor class.

### 4.1 Cardiovascular and thrombotic risk associated with C5 inhibition in MG

To date, evidence supporting the use of eculizumab and ravulizumab in MG has been derived primarily from randomized trials and extension studies that focused on neuromuscular efficacy and general safety (9, 12, 26).

Cardiovascular events in these studies were reported only as aggregated adverse events and were not evaluated as predefined outcomes. Similarly, recent real-world and registry-based studies have provided important insights into treatment persistence, effectiveness, and tolerability of C5 inhibitors in MG (13, 15, 16, 18, 19, 27). None of these studies were designed to evaluate cardiovascular outcomes using standardized endpoint definitions or time-to-event methodology. Our findings therefore extend the current evidence by demonstrating that C5 inhibition in MG is associated with an increase in clinically relevant cardiovascular and thrombotic events with a pattern suggesting a predominance of arterial rather than venous thrombotic events, which may indicate a contribution of atherosclerotic and inflammatory vascular mechanisms rather than isolated venous thromboembolism.

The increase in the composite MACE endpoint appeared to be driven mainly by arterial cardiovascular events rather than venous thromboembolism, suggesting that inflammatory and endothelial mechanisms may play a larger role than classical venous thrombosis pathways.

Similar thrombotic and cardiovascular complications have been described in other complement-mediated diseases treated with C5 inhibitors (6), including PNH and aHUS, where complement-mediated endothelial injury, inflammation, and thrombosis contribute to vascular events (28–30). These findings suggest that the cardiovascular risk observed under complement inhibition may reflect complex interactions between complement activity, inflammation, endothelial function (31), and baseline cardiovascular risk rather than a direct prothrombotic drug effect alone. From a clinical perspective, the observed increase in cardiovascular morbidity but reduction in overall mortality suggests that improved disease control and reduced myasthenic and respiratory complications may outweigh cardiovascular risk during the first year of treatment, but cardiovascular risk assessment and monitoring remain important in patients receiving long-term complement inhibition.

Importantly, these associations were consistent across analytical approaches. Propensity-matched analyses identified increased odds of AKI, thrombotic disorders, arrhythmias, and MACE, while Kaplan-Meier analyses confirmed higher hazards for AKI and thrombotic events and a sustained divergence in MACE incidence over time. The concordance of these findings supports their robustness and reduces the likelihood that they are explained solely by baseline differences or surveillance bias.

We performed an additional subgroup analysis stratified by dyslipidaemia within the C5 inhibitor cohort. However, after PSM the subgroup sizes and event counts were too small to allow reliable statistical comparison. Importantly, dyslipidaemia and other cardiovascular risk factors were already included in the PSM, resulting in well-balanced cohorts and reducing confounding by baseline atherosclerotic risk. Therefore, the observed increase in thrombotic events is unlikely to be explained solely by differences in baseline atherosclerotic risk and may instead reflect a multifactorial interaction between cardiovascular risk factors, systemic inflammation, and complement inhibition.

### 4.2 Mechanistic context for renal and cardiovascular safety associated with C5 inhibition

Complement C5 inhibition may influence cardiovascular and renal safety through interconnected infectious, thrombo-inflammatory, vascular, and myocardial mechanisms. Terminal complement blockade increases vulnerability to severe bacterial infections, including bacteraemia or urinary tract infections, which can lead to infection-associated AKI (32, 33). In parallel, experimental and translational data indicate that local complement signalling within the kidney contributes to anti-inflammatory and tubular repair mechanism following injury. Thus, C5 blockade may impair adaptive recovery after ischemic or toxic insults and lead to persistent or more severe AKI (34).

In addition, complement activation is a key driver of endothelial injury and microvascular thrombosis in complement-mediated thrombotic microangiopathy (31), which frequently involves the kidney and may lead to AKI through microangiopathic and thrombotic mechanisms (29, 30). Complement-mediated thrombotic microangiopathy and aHUS are well-established examples in which dysregulated complement activation leads to endothelial damage, thrombosis, and renal injury (14). Furthermore, complement activation has been implicated more broadly in the pathophysiology of AKI, where complement-mediated inflammation and endothelial dysfunction contribute to tubular injury and impaired renal recovery (34, 35). Importantly, eculizumab has been used in catastrophic antiphospholipid syndrome, a severe thrombotic microangiopathic condition characterized by widespread thrombosis, multiorgan failure, and AKI, further supporting the link between complement activation, thrombosis, and renal injury (28, 36).

Taken together, these mechanisms suggest that renal injury observed in patients receiving C5 inhibition may be related not only to infection-associated kidney injury but also to complex interactions between complement regulation, endothelial function, thrombosis, and renal microvascular injury.

The thrombotic risk during C5 inhibition appears to arise mainly from transient loss of effective terminal complement control rather than from a direct prothrombotic drug effect. During periods of insufficient complement blockade, membrane attack complex formation can occur, leading to release of intracellular mediators such as ADP that trigger platelet activation and thrombosis, whereas sustained C5 inhibition prevents this pathway (37). Clinically, this mechanism is reflected in breakthrough haemolysis, which is often triggered by complement-amplifying conditions such as infection or surgery. These episodes are temporally associated with thrombotic complications and highlight incomplete or transient complement suppression as a proximate risk factor (38). Consistent with this model, long-term data from PNH demonstrate that sustained and stable terminal complement inhibition, particularly with ravulizumab, is associated with low rates of MACE, supporting the concept that thrombosis during C5 inhibition is largely driven by episodic complement breakthrough rather than chronic drug-related toxicity (39).

Finally, C5 inhibition may modulate the risk of arrhythmias and MACE through vascular and myocardial pathways, including C5a-C5aR1-mediated endothelial dysfunction, glycocalyx degradation, impaired nitric oxide bioavailability, and complement-driven myocardial inflammation and remodelling, which together create an ischemic and electrophysiologically vulnerable substrate for arrhythmias and MACE (31, 37, 40–42).

### 4.3 Survival benefit despite increased cardiovascular morbidity

A notable and clinically important observation was the improvement in overall survival among C5-treated patients, despite higher rates of cardiovascular and thrombotic complications. Time-to-event analyses demonstrated significantly lower mortality with C5 inhibition, whereas odds-based analyses showed a consistent but non-significant trend in the same direction. This pattern suggests that during the first year of treatment, C5 inhibition offers a survival benefit that is probably driven by improved disease control and fewer myasthenic and respiratory complications. The potential benefits of this approach may be sufficient to outweigh the observed increase in cardiovascular morbidity that has been documented in the early treatment period (12, 18). This finding underscores the importance of evaluating cardiovascular safety independently of mortality.

### 4.4 Disease context and comparison with other complement-mediated disorders

The cardiovascular risk observed in this study should be interpreted in the broader context of MG as a systemic autoimmune disorder characterized by chronic immune activation. Supporting this concept, Huang et al. demonstrated increased expression of CD95 (Fas-Receptor) on CD4^+^ effector-memory T cells in MG patients, correlating with disease severity and a pro-inflammatory immune phenotype (43).

In addition, recent studies have demonstrated elevated circulating inflammatory cytokines and systemic immune activation in patients with MG, supporting the concept that MG represents a chronic inflammatory autoimmune disease rather than a purely neuromuscular disorder (4). Chronic as well as acute inflammation is known to promote endothelial dysfunction, platelet activation, and prothrombotic pathways, and autoimmune diseases in general are associated with increased cardiovascular and thrombotic risk (5, 44, 45). Therefore, patients with MG may already have an elevated baseline cardiovascular and thrombotic risk, independent of complement inhibition, and C5 inhibition may interact with this inflammatory milieu rather than representing an isolated prothrombotic drug effect (4–7).

Importantly, this disease context distinguishes MG from other complement-mediated conditions. In PNH and aHUS, complement inhibition directly mitigates complement-driven endothelial injury and haemolysis, resulting in a net reduction of thrombotic risk (22, 46, 47). Consequently, systemic C5 blockade in MG may not provide vascular protection. Rather, it may alter the immune-inflammatory balance without providing any endothelial benefit. This disease-specific context may explain why cardiovascular safety signals are observed in patients with MG receiving complement inhibition. Cardiovascular risk should therefore be assessed directly in this population rather than extrapolated from other complement-mediated disorders (22, 46, 47).

Long-term data from PNH, including pooled analyses of recent phase III trials (NCT02946463 and NCT03056040) with up to six years of follow-up, demonstrate that ravulizumab is associated with low and stable rates of major adverse vascular events, reported at approximately 0.7-1.4 events per 100 patient-years (48). These cohorts included both C5-inhibitor-naïve patients and those previously treated with eculizumab that switched to ravulizumab-treatment. Those data represent the most comprehensive long-term vascular safety dataset for ravulizumab to date and point to a more favourable cardiovascular profile for ravulizumab which was also confirmed by our analyses.

Consistently, randomized phase III comparisons of ravulizumab and eculizumab did not demonstrate excess thrombotic or cardiovascular events with ravulizumab during controlled treatment periods. Complementing these data, pharmacovigilance analyses of spontaneous adverse event reports suggest broadly similar event-rates for eculizumab and ravulizumab, without any excessive cardiovascular safety risks (18). Mechanistic reviews further support that sustained terminal complement inhibition reduces haemolysis-driven inflammation and thrombosis, while noting that intermittent or incomplete complement suppression may permit residual inflammatory or prothrombotic activity (49).

### 4.5 Differential cardiovascular safety of ravulizumab and eculizumab

A key clinically finding of this study is the lower incidence and hazard of MACE associated with ravulizumab compared with eculizumab. Prior randomized trials and real-world studies have demonstrated comparable neuromuscular efficacy and general tolerability between the two agents but were not designed to assess cardiovascular outcomes (13, 15, 16, 18–20, 27). Our analysis therefore provides novel evidence suggesting differential cardiovascular safety within the C5 inhibitor class. However, it should be noted that the composite endpoint MACE is not uniformly defined across studies and may thus include a range of heterogeneous clinical conditions, requiring cautious interpretation of the observed difference between eculizumab and ravulizumab.

Although mechanistic conclusions cannot be drawn from our observational data, ravulizumab’s more stable pharmacokinetic profile and reduced complement breakthrough may contribute to a more consistent immunologic milieu, potentially mitigating inflammatory or thrombotic perturbations. From a clinical perspective, these findings support consideration of cardiovascular risk when selecting long-term C5 inhibitor therapy in MG.

### 4.6 Strengths and limitations

Strengths of this study include the large sample size, use of a real-world data, rigorous PSM, and the integration of odds-based and time-to-event analyses. This design enabled the evaluation of infrequent but clinically important cardiovascular outcomes that are not accessible in randomized trials.

This study is limited by its observational design, reliance on coded diagnoses, and the absence of granular data on MG severity, antibody titters, and detailed cardiovascular risk factors. Therefore, confounding effects cannot be fully excluded. However, the use of large, PSM cohorts and complementary odds-based and time-to-event analyses mitigates baseline imbalance and supports the robustness of our findings. Limited information on treatment dynamics and complement breakthrough reflects real-world care and captures the net clinical effect of sustained C5 inhibition. Follow-up was restricted to one year, but this interval is clinically meaningful for identifying early cardiovascular safety signals in a rare disease population, while longer-term effects should be investigated in future analyses.

### 4.7 Clinical implications

Taken together, our findings demonstrate that C5 inhibition in generalized MG is associated with improved survival but increased cardiovascular and thromboembolic morbidity, highlighting a complex safety profile that has not been previously characterized. The observed lower MACE risk with ravulizumab compared with eculizumab suggests meaningful heterogeneity within the C5 inhibitor class and has direct implications for treatment selection and long-term risk management. These results support routine cardiovascular risk assessment and monitoring in patients with MG treated with complement inhibitors and highlight the need to incorporate cardiovascular safety into therapeutic algorithms and future guidelines.

## Data Availability

The original contributions presented in the study are included in the article/supplementary material. Further inquiries can be directed to the corresponding author.
